# Corrosion of an Additively Manufactured Ti6Al4V Alloy in Saline and Acidic Media

**DOI:** 10.3390/ma17030712

**Published:** 2024-02-02

**Authors:** Hugo Mora-Sanchez, Miguel Collado-Vian, Marta Mohedano, Raúl Arrabal, Endzhe Matykina

**Affiliations:** 1 Departamento de Ingeniería Química y de Materiales, Facultad de Ciencias Químicas, Universidad Complutense de Madrid, 28040 Madrid, Spain; migcolla@ucm.es (M.C.-V.); mmohedan@ucm.es (M.M.); rarrabal@ucm.es (R.A.); e.matykina@quim.ucm.es (E.M.); 2 CIDETEC, Basque Research and Technology Alliance (BRTA), Po. Miramón 196, 20014 Donostia-San Sebastián, Spain

**Keywords:** additive manufacturing, selective laser melting, titanium, heat treatment, acid rain, seawater, corrosion, passive layer

## Abstract

The present work aims to provide corrosion performance data for an additively manufactured Ti6Al4V alloy in saline and polluted environments. The as-received additively manufactured material underwent heat treatment at 850 °C for 3 h to transform the acicular α’ microstructure into a lamellar α microstructure. Comparative corrosion assessments were conducted between the heat-treated substrates, the as-received condition, and a conventionally mill-annealed alloy. Potentiodynamic polarization experiments were carried out in saline (3.5 wt.% NaCl) and acid aqueous media ((NH_4_)_2_SO_4_ containing Harrison’s solution). The corrosion performance of additively manufactured substrates matched or surpassed that of the conventional alloy in Harrison’s solutions while remaining inferior in saline medium, despite forming a thicker passive film. Overall, the XY plane showed better corrosion performance, particularly after the elimination of the acicular α’ martensite by the applied heat treatment. The results also suggested that the presence of the coarse β phase was beneficial in 3.5 wt.% NaCl solution and detrimental in Harrison’s solutions, more so in acidified and fluorinated conditions.

## 1. Introduction

There is little doubt nowadays that Ti6Al4V is a well-established alloy in the aeronautical and biomedical fields due to its weight-to-strength ratio, corrosion resistance, and biocompatibility, among other properties [1,2]. On the other hand, additive manufacturing (AM) techniques have emerged as the most suitable option for fabricating complex–shape components [3]. For instance, turbine blades that may require internal cooling channels or orthopedic appliances [4,5,6,7], which can be fabricated by AM with a near-net shape, require fewer post-machining operations than traditional manufacturing [8]. Henceforth, the fabrication of Ti6Al4V by AM techniques has become of great interest to the research community [9,10].

Ti6Al4V manufactured by laser powder bed fusion (L-PBF) technologies, such as selective laser melting (SLM) and direct melting laser sintering (DMLS), presents microstructures characterized by (1) anisotropic macrotextures in the building orientation, prior-beta elongated grains in the XZ planes, and a chessboard pattern in the XY plane [11]; and (2) the predominant presence of the non-equilibrium martensitic α’ phase in contrast to conventional α + β Ti6Al4V [12] as a consequence of the fast cooling of the β phase during the AM process (>410 °C/s, [13]). The anisotropy between planes also produces higher ultimate tensile strength (UTS) and yield strength (YS) in the parallel plane and larger elongations in the perpendicular plane [14]. The mechanical properties are henceforth determined by this microstructure. The deformed lattice structures and high density of dislocations of the non-equilibrium martensitic α’ provide higher strength with respect to α + β microstructures at the expense of ductility.

Regarding the electrochemical response, electrochemical anisotropy has been reported in HCl for Ti-6Al-4V produced by SLM, and the inferior corrosion resistance of the XZ-plane was ascribed to the presence of more α’ martensite and less β-Ti phase in the microstructure [15]. More importantly, the metastable α’ microstructure has been shown to reduce the corrosion resistance of AM-ed Ti6Al4V with respect to traditionally manufactured materials (lamellar α + β and equiaxed α) since the distorted phase represents a higher energetic state [16]. On the other hand, electrochemical studies in solutions such as simulated body fluid (SBF), phosphate buffer solution (PBS), and Hanks’ solution have shown that AM-ed Ti6Al4V might present similar or even superior corrosion resistance than the wrought counterpart [11,17,18,19]. This indicates that the AM condition might be safer for biomedical applications than for transport applications.

Thermal treatment strategies represent an important part of the research efforts to optimize the mechanical performance of AM-ed appliances [20]. Thermal treatments are used to relieve internal stresses, homogenize the microstructure, and gain ductility. These treatments imply the transformation of the martensitic acicular α’ phase.

For thermal treatments above ~400 °C and below the β transus temperature (T_β_, ~995 °C), the α’ phase decomposes into α, due to the diffusion of V, which promotes the formation of β at the α boundaries [21]. The size of α laths increases with the heat treatment temperature, and it may become a fully lamellar microstructure if the temperature is high enough [22]. According to Zhang et al. [20], SLM Ti6Al4V heat treated for 2 h at 800 and 850 °C, followed by furnace cooling, led to elongations to failure larger than in the as-built condition (~21% vs. 13%) and comparable to the as-forged one (21%), while the yield strength (~1100 MPa) and UTS (~1400 MPa) decreased with respect to the as-built condition (1292 and 1603 MPa, respectively) but were superior to the ones of the as-forged alloy (783 and 1347 MPa). Also, the study concluded that higher temperatures led to a further reduction in the yield strength and UTS.

For thermal treatments at a temperature above T_β_, α’ transforms into β, which is then partially transformed into α during furnace cooling, obtaining a final coarse lamellar α + β microstructure, which, in turn, significantly reduces the yield strength and UTS [22].

Indeed, microstructural changes not only affect mechanical performance but also electrochemical behavior. Heat treatments between 500 and 600 °C [22,23] barely presented changes neither in microstructure (fine acicular α’) nor electrochemical response with respect to the as-built samples. Yang et al. [22], Ettefagh et al. [21], and Dai et al. [23] studied the effect of annealing on the corrosion resistance of SLM Ti6Al4V in 3.5 wt.% NaCl. The thermal treatments were carried out for 2 h at 750 °C, 800 °C, and 850 °C, respectively, followed by furnace cooling. For the thermal treatments carried out at 750 and 800 °C [21,22], the E_corr_ (corrosion potential, V), i_corr_ (corrosion current density, μA/cm^2^), and i_p_ (passive current density, μA/cm^2^) approximated the values of the traditionally manufactured samples used as a reference. In the case of 850 °C [23], the material presented slightly higher corrosion and passive current densities. For thermal treatments above the T_β_, the electrochemical response was similar to or worse than the non-treated sample [22,23,24,25].

The improvement observed for the samples treated at 750 and 800 °C was mainly assigned to the elimination of the defective α’ in favor of α. In addition, it was also proposed that the increased content of β would also improve the corrosion performance. The role of the β phase is always discussed since it is known to reduce the corrosion rate of α + β alloys compared with α alloys. Dai et al. [15] concluded that the better corrosion performance of the XY plane in SLM Ti6Al4V was due to the higher content of β phase against α’. However, it appears that with the α’ → α transformation, defects and grain size play a more important role. The authors in [23] determined that even though the content of β increased with the treatment temperature, the coarsening of the alpha laths decreased the corrosion resistance. In agreement, Yang and co-workers [22] concluded that grain size was the second determining factor for corrosion protection following the phase type (the α phase was preferred to α’, and β was preferred to α). The researchers also performed a thermal treatment above T_β_, in addition to the sub-T_β_ (800 °C). Although the content of the β phase was drastically increased (lamellar α + β), the corrosion performance was found worse than the sample treated at 750 °C (fine α lamellae + β at boundaries). They claimed that this was due to the coarser α + β microstructure.

Gong et.al. [26] studied the effect of the building orientation in Ti6Al4V fabricated by electron-beam manufacturing (EBM). Due to the high in-process temperatures (600–700 °C), an as-built α lamellar microstructure with β distributed within the α grain boundaries was produced. The 45° sample presented the worst corrosion performance and had the largest density of grain boundaries and the highest content of β phase. On the contrary, the 0° sample, which had the lowest grain boundary density and β content, presented the best corrosion performance. It has also been proposed that given the difference in electrochemical properties between α and β phases, microgalvanic pairs may develop, leading to local corrosion.

Henceforth, it could be concluded that, from the corrosion performance point of view, Ti6Al4V produced by AM processes that lead to an as-built martensitic α’ microstructure should be subjected to thermal treatments below T_β_ at a temperature that allows the α’ to α transformation, avoiding excessive α lath growth and β segregation. According to the available literature, suitable temperatures are in the range between 750–850 °C.

The number of works regarding the corrosion performance of AM Ti6Al4V and the effect of post-AM heat treatments is relatively limited. In the present work, we compare the corrosion performance of heat-treated DMLS Ti6Al4V with the as-built condition and a hot-rolled mill-annealed α + β Ti6Al4V. In addition, both XY and XZ orientations were studied. Most of the published reports were focused on transport-related environments such as HCl, H_2_SO_4_, and mostly NaCl [27]. The effect of the so-called acid rain, resulting from the presence of NOx, SO, and H_2_S, among other pollutants in the air, has raised concerns in the construction [28,29] and aviation industries [30]. However, there is a general lack of literature dealing with the electrochemical response of Ti and its alloys in acid rain conditions [31]. Henceforth, in this work, we studied the electrochemical response of as-received and heat-treated DMLS Ti6Al4V, in both NaCl and simulated acid rain solution, by means of potentiodynamic polarization (PDP) and electrochemical impedance spectroscopy (EIS).

## 2. Materials and Methods

### 2.1. Ti6Al4V Substrates

**Figure 1:**
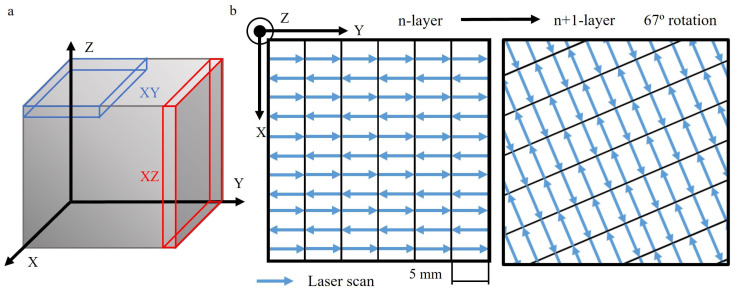
Additive manufacturing L-PFB alloy. (**a**) Specimens extracted in the studied orientations. (**b**) Scan strategy from the n-layer to the subsequent n + 1-layer.

The L-PBF Ti6Al4V alloy was studied in both the as-received condition (AR) and after heat treatment (HT). In both conditions, specimens were examined in both XY and XZ orientations (Figure 1a). Specimens were obtained from dense 30 mm × 30 mm × 30 mm cubes, which were produced using an M280 EOS Direct Metal Laser Sintering system (EOS GmbH, Krailling, Germany) by Fundación Idonial (Gijón, Spain). The particle size of the powder feedstock varied in the range between 20 and 63 μm. The laser used had a power of 280 W and a spot size of 0.07 mm, scanning each layer with a thickness of 0.03 mm. The laser traveled in alternating scans along 5 mm stripes with a hatch spacing of 0.09 mm at a speed of 1200 mm/s, covering the entire printing area (Figure 1b). This pattern was rotated by 67° for each subsequent layer to minimize residual stresses.

After the manufacturing process, the cubes underwent stress relief thermal treatment at 650 °C for 3 h, constituting the as-received (AR) condition. Another cube was subjected to an additional heat treatment at 850 °C for 3 h to transform the microstructure, representing the heat-treated (HT) condition. As a control (C) specimen for comparison, a hot-rolled mill-annealed Ti6Al4V conventional alloy was chosen.

For the electrochemical characterization of the substrates, the specimens were ground to 4000P SiC and subsequently etched for 20 s in Kroll solution composed of 48 mL distilled H_2_O, 40 mL HNO_3_, and 12 mL HF.

### 2.2. Substrates Characterization

Microstructural characterization of the substrates involved metallographic preparation through successive SiC abrasive papers ranging from P120 to P4000 grade. This was followed by 3 μm and 1 μm diamond polishing, concluding with a final polishing step with OPS colloidal silica. To enhance microstructure contrast, the polished surface underwent etching in Kroll solution. Optical microscopy (Leica DMi8, Leica Microsytems now belongs to Danaher, Washington, DC, USA) was employed to capture images of the microstructure. A JEOL 6400 JSM (Tokyo, Japan) scanning electron microscope (SEM) equipped with an Oxford Link energy dispersive X-ray (EDS) microanalysis spectrometer was used for detailed morphological and semi-quantitative compositional characterization.

The crystallographic phases of the substrates, both before and after thermal treatments, were identified using X-Ray Diffraction (XRD). Diffractograms were acquired in a Bragg–Brentano configuration with Cu as the cathode (Cu Kα = 1.54056 Å). Scans were obtained from 10° to 90° (2θ) at a step size of 0.04°, with each step lasting 15 s, using a PANalytical X’Pert diffractometer (Malvern Panalytical, Almelo, The Netherlands).

### 2.3. Electrochemical Characterization

#### 2.3.1. Potentiodynamic Polarization

Potentiodynamic polarization (PDP) experiments were conducted in the following saline and acidic aqueous media:

1. A total of 3.5 wt.% NaCl;
2. Harrison’s solution (Harr) 0.05 wt.% NaCl and 0.35 wt.% (NH_4_)_2_SO_4_ (pH 6.2–6.5);
3. Modified Harrison’s solution with a pH of 4 (Harr4);
4. Modified Harr4 with the addition of 0.12 mg/dm^3^ F^−^ (Harr4-F).

Prior to PDP, the specimens were immersed in the testing media for one hour, during which the open circuit potential (OCP) was measured. The PDP tests were performed from −0.5 V to 3.5 V relative to the OCP at a scan rate of 0.5 mV/s, with a minimum of 1 cm^2^ exposed to the medium at room temperature. The electrochemical tests were carried out in a three-electrode configuration, utilizing an Ag/AgCl reference electrode and a graphite rod as a counter electrode. The experiments were conducted using a Gamry Interface 1010E Potentiostat/Galzanostat/ZRA (Gamry Instruments, Warminster, PA, USA), and two specimens per condition were used.

#### 2.3.2. Electrochemical Impedance Spectroscopy (EIS)

EIS experiments were conducted in a 3.5 wt.% NaCl aqueous solution for both the C and AMXY-HT substrates, using the same electrode configuration and equipment utilized in the PDP tests. The frequency was swept in the range between 10^5^ and 0.01 Hz, acquiring 10 points per decade. The amplitude of the sinusoidal signal was 10 mV. The resulting impedance spectra were subjected to fitting using ZView version 3.1c software, employing equivalent electrical circuits while maintaining chi-squared values below 0.01 to ensure the goodness of the fit. For each individual parameter of the equivalent circuits, the errors were kept below 5%. Prior to EIS measurements, specimens were immersed in the saline medium for 1 h to record the OCP. EIS measurements were performed at 0, 0.5, 2, and 3 V relative to the OCP, with a new surface used for each experiment.

## 3. Results and Discussion

### 3.1. Substrate Materials

**Figure 2:**
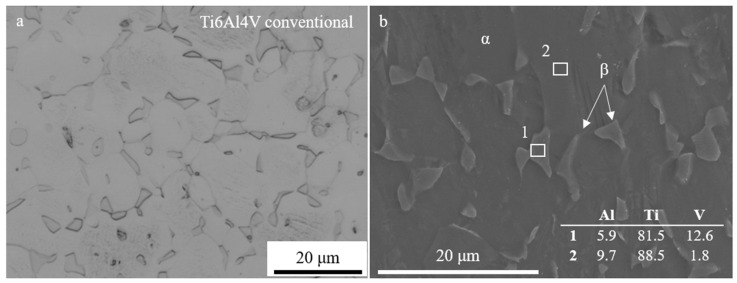
Optical microscopy (**a**) and scanning electron microscopy (**b**) images of the conventional Ti6Al4V alloy showing the α matrix and β grains. Numbered boxes in (**b**) correspond to locations of EDS measurements shown in the table in image (**b**).

**Figure 3:**
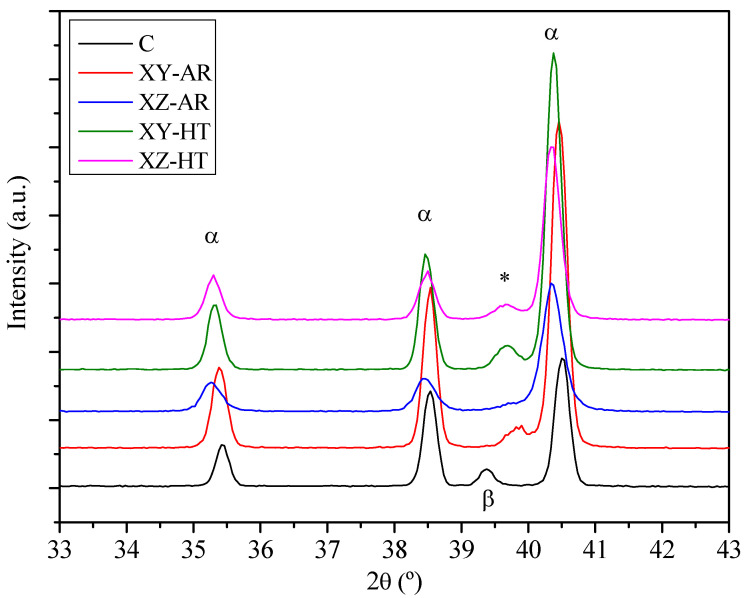
X-ray diffractograms of the conventional (C) and AM substrates before (XY/XZ-AR) and after the heat treatment (XY/XZ-HT). Labels stand for: α, Ti alpha phase; β, Ti beta phase; *, indicates peaks assigned to V-rich phase.

The conventional alloy showed a microstructure consisting of equiaxed grains (Figure 2a,b), corresponding to an α matrix and β grains according to the XRD diffractograms (Figure 3).

**Figure 4:**
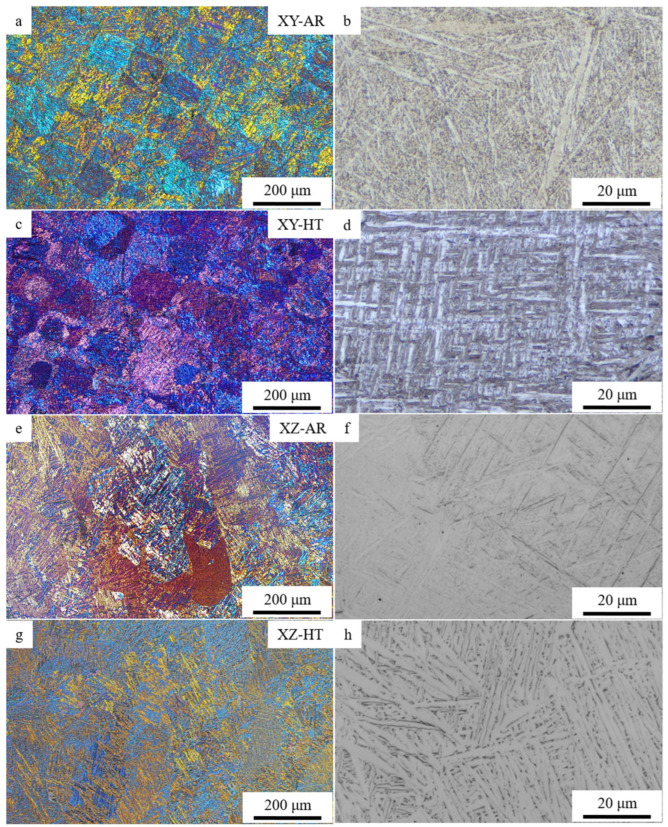
Optical microscopy images of additively manufactured samples: (**a**,**b**) XY-AR; (**c**,**d**) XY-HT; (**e**,**f**) XZ-AR; (**g**,**h**) XZ-HT.

Figure 4 shows the optical micrographs of the XY-AR (a and b), XY-HT (c and d), XZ-AR (e and f), and XZ-HT (g and h) samples. Images in polarized light show that the XY-AR (Figure 4a) and XZ-AR (Figure 4e) samples presented the typical chessboard pattern and columnar structure, respectively, of L-PBF Ti6Al4V alloys. Such microstructures were distorted after the heat treatment (XY-HT Figure 4c and XZ-HT Figure 4g, respectively). Higher magnification images showed that the characteristic α’/α needles (Figure 4b,f) transformed after the heat treatment into thicker α lamellas (Figure 4d,h).

**Figure 5:**
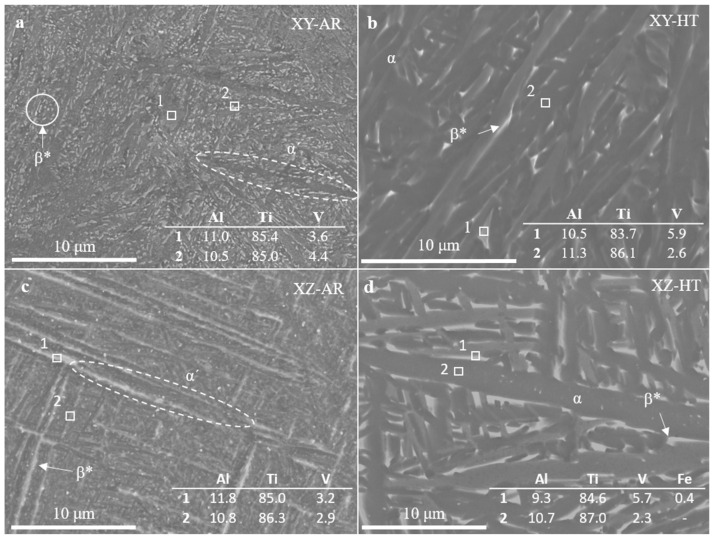
Scanning electron microscopy images of additively manufactured samples: (**a**) XY-AR; (**b**) XY-HT; (**c**) XZ-AR; (**d**) XZ-HT. Numbered boxes correspond to the locations of EDS measurements. The composition is given in the tables within the images in %. Labels stand for: α, Ti alpha phase; β, Ti beta phase; β*, beta aggregates.

**Figure 6:**
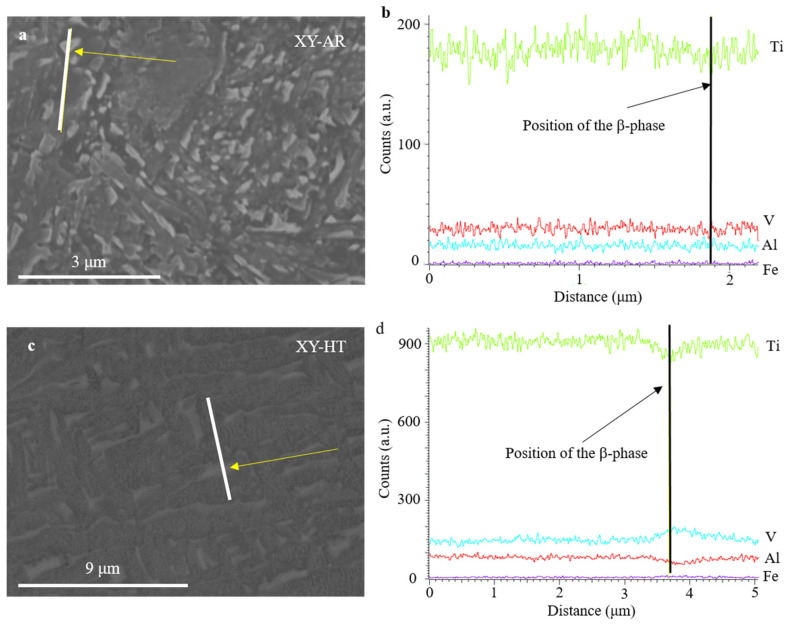
Scanning electron microscopy images (**a**,**c**) and EDX line scans (**b**,**d**) of additively manufactured samples XY-AR (**a**,**b**) and XY-HT (**c**,**d**). The line scans were taken as indicated by the white lines in the SEM images across β-phase particles indicated by yellow arrows.

SEM images and EDS measurements in Figure 5 show the microstructural transformation after the heat treatment in better detail. The XY-AR (Figure 5a) and XZ-AR (Figure 5c) samples presented a fine microstructure with a darker phase, corresponding to the α’/α needles, and a brighter phase with a slightly higher content of V (spectrum 2 in Figure 5a and spectrum 1 in Figure 5c). After the heat treatment, both XY-HT and XZ-HT (Figure 5b and d, respectively) showed thicker α lamellas (darker phase in SEM images), as well as a larger brighter phase with higher content of V (spectrum 1 in Figure 5b and spectrum 1 in Figure 5d). Figure 6 shows EDS line scan measurements on the XY-AR (Figure 6a,b) and XY-HT (Figure 6c,d) samples. The line scans were taken along the darker α’/α phase and across brighter particles. In the case of the XY-AR sample, the line scan in Figure 6b did not show a significant variation of composition across the brighter particle. In contrast, for the XY-HT sample, Ti and Al contents decrease at the brighter particle, while the V line shows a peak.

XRD diffractograms revealed that AR and HT conditions, for both XY and XZ orientations, (Figure 3) showed peaks corresponding to the martensitic α’ needles and/or α lamellas. The XY-AR and XZ-AR samples presented a shoulder near 40°, which was noticeably more pronounced in the XY-AR sample. The XY-HT and XZ-HT samples presented wide peaks nearer to 39.5°. These peaks can be assigned to the formation of a V-rich phase (marked as * in Figure 3), associated with the initial formation of small β aggregates at the α grain boundaries [20] even after a thermal treatment at 600 °C for 2 h [17]. Therefore, for the present investigation, it can be concluded that (i) traces of β aggregates within the grain boundaries of α’/α needles at the AR condition (650 °C for 3 h) were found and (ii) higher temperatures facilitated the diffusion of V toward the grain boundaries of α lamellas, leading to the growth of the β aggregates. In the following, this will be referred to as β*.

### 3.2. Electrochemical Characterization

**Figure 7:**
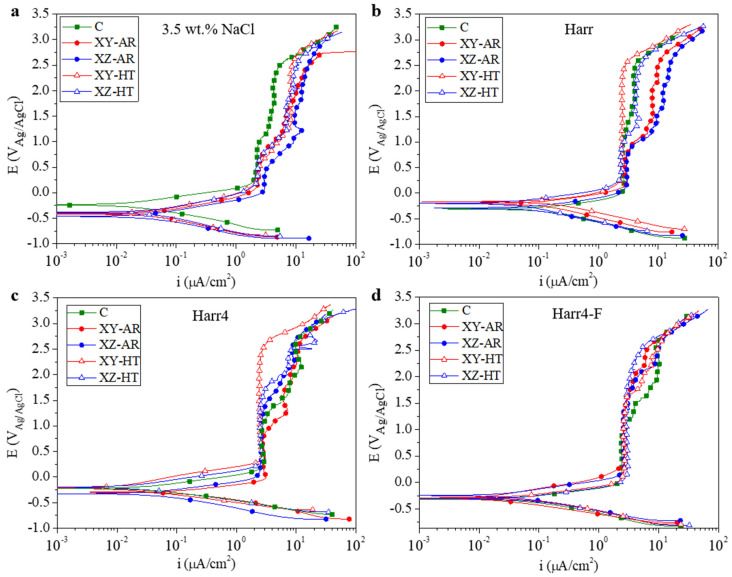
Potentiodynamic polarization curves in (**a**) 3.5 wt.% NaCl; (**b**) Harrison’s solution; (**c**) modified Harrison’s solution with a pH of 4; and (**d**) modified Harr4 with the addition of 0.12 mg/dm^3^ F^−^ (Harr4-F).

**Figure 8:**
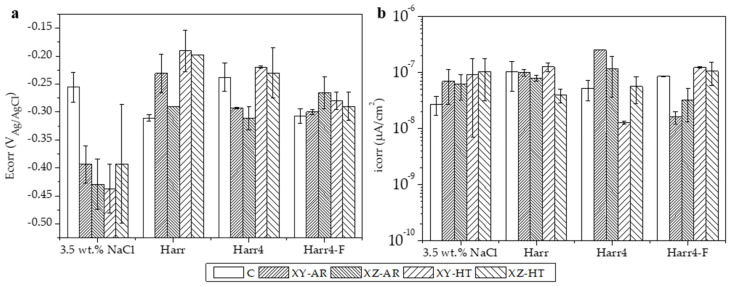
(**a**) Corrosion potential (Ecorr) and (**b**) current density (icorr) values of the specimens tested in all four media. Values were extracted from curves in Figure 7.

The potentiodynamic polarization curves (PDP) for all the studied substrates in the 3.5 wt.% NaCl, Harr, Harr4, and Harr4-F solutions are presented in Figure 7 and are labeled from (a) to (d), respectively. The values of corrosion potential and current density (E_corr_ and i_corr_, respectively) extracted from the PDP curves through Tafel fitting of the cathodic branch are depicted in Figure 8. In all cases, the substrates presented PDP curves with a similar shape, demonstrating passive behavior in the anodic branch, particularly above 0 V (Figure 7).

#### 3.2.1. Behavior at the OCP Region

The E_corr_ values showed distinct behavior in the 3.5 wt.% NaCl medium, with the C alloy being significantly nobler (E_corr_ approximately −0.25 V) than the AM-ed alloys (−0.45–−0.4 V). In the unmodified Harrison’s solution (Harr pH 6.2–6.5), the C alloy became less noble and had a greater i_corr_ in comparison with the 3.5 wt.% NaCl solution. On the other hand, the AM-ed alloys presented a higher E_corr_ (>−0.3 V) than the C alloy while having similar i_corr_. Under acidic conditions in the Harr4 solution, the C, XY-HT, and XZ-HT samples presented similar E_corr_ (−0.25–−0.2 V) and i_corr_ values, while the XY-AR and XZ-AR presented lower E_corr_ and higher i_corr_. Finally, in the harsher Harr4-F solution, all the specimens presented similar E_corr_ values (−0.3–−0.25 V). In terms of i_corr_, the XY-AR and XZ-AR samples presented the lowest currents while the C, XY-HT, and XZ-HT samples showed similar values.

From these results, it can be concluded that in the 3.5 wt.% NaCl solution, the additively manufactured alloy, in both AR and HT conditions, had lower corrosion resistance than the conventional alloy. However, in the Harr, Harr4, and Harr4-F solutions, the behavior of the AM-ed alloys was often comparable to or even better than that of the C alloy.

#### 3.2.2. Behavior under Anodic Polarization

In all cases, a passive region at approximately 2 μA/cm^2^ was observed in the anodic branch of the PDP curves (Figure 7) at voltages above 0 V. Beyond 0.5 V, the behavior varied for each alloy and medium.

In the 3.5 wt.% NaCl solution (Figure 7a), the C sample had a first passive segment at 2 μA/cm^2^ up to approximately 1 V, where a second segment initiated at 4 μA/cm^2^ up to 2.5 V. In the case of the AM-ed alloys, the first passive segment at 2 μA/cm^2^ (3 μA/cm^2^ for the XZ-AR) ended at a lower potential of 0.5 V. Also, the current density increased up to a higher value of ~8 μA/cm^2^.

In the Harr solution (Figure 7b), the XY-HT alloy had the best performance, with a single passive segment at 3 μA/cm^2^ that extended up to 2.5 V. In contrast, the C, XZ-HT, and AR samples reached values between 4 and 10 μA/cm^2^.

In the Harr4 medium (Figure 7c), the XY-HT substrate still showed the best performance (2 μA/cm^2^ up to 2.5 V). It is worth noting that the lower pH in this case negatively affected the C alloy, thus becoming one of the worst performers.

Finally, for the Harr4-F medium (Figure 7d), the AM-ed alloys presented a passive segment at 2 μA/cm^2^ that extended up to higher potentials (>1.5 V) than that of the C sample (1 V).

The constant current density regions, denoted as first and second segments, can be related to the formation of a passive film that is less protective at higher potentials, as evidenced by the higher current density values. Subsequently, the continuous rise in current density at potentials above 2.5 V is associated with the continuous dissolution of the passive film.

Therefore, the C alloy formed a passive surface that was relatively stable in the 3.5 wt.% NaCl and Harr solutions, and it was more susceptible to dissolution in the Harr4 and Harr4-F solutions. In contrast, the XY-HT and XZ-HT samples had an improved passive behavior in the Harr solution and subsequent modifications, especially for the XY-HT sample, indicating certain anisotropy in the heat-treated AM alloy.

**Figure 9:**
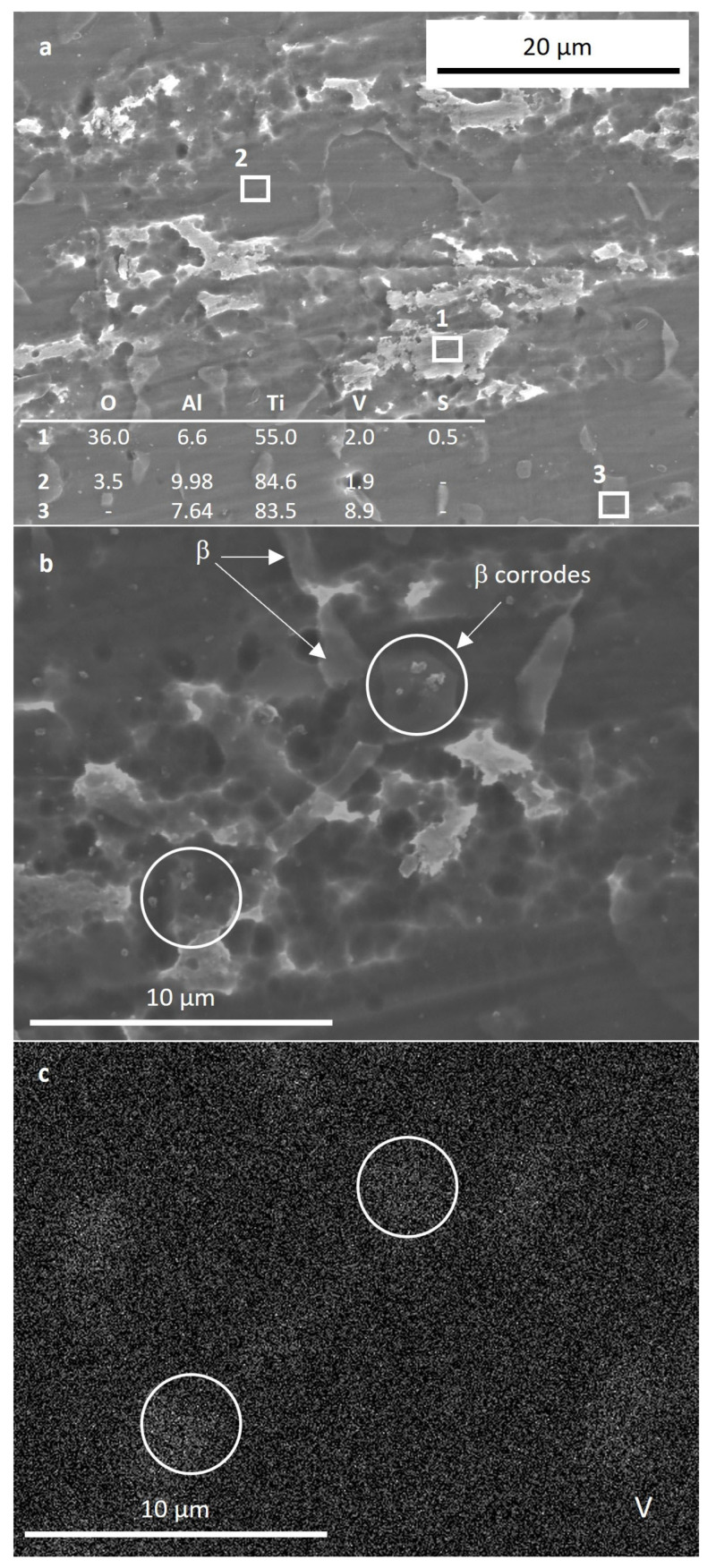
SEM images of the C sample after 1 h immersion in Harr solution polarized at 2.15 V. (**a**) overview of the sample surface showing the corrosion of all the phases; (**b**) high magnification image of corroded β phase; (**c**) V element EDS map of the region shown in (**b**).

In order to study in more detail the anodic behavior in the Harr solution, new samples for each condition were immersed in this solution and polarized up to 2.15 V, corresponding to the second passive region. The samples were maintained at this voltage for 1 h. SEM images of the C alloy after this experiment are shown in Figure 9. Figure 9a revealed the thickening of the oxide film on both α and β grains. Corrosion appeared more severe at α grains, which presented rounded pits. The white rougher oxide film formed on certain α grains (spectrum 1 in Figure 9a, low V content), as opposed to other α locations, which appeared darker and smoother (spectrum 2 in Figure 9a), is possibly related to α basal planes [32]. The formation of this film may explain the second passivation segment of the C sample in Figure 7b. Although, to a less significant degree, β-phase grains also underwent corrosion, as can be seen at the rough V-rich locations shown in Figure 9b,c.

**Figure 10:**
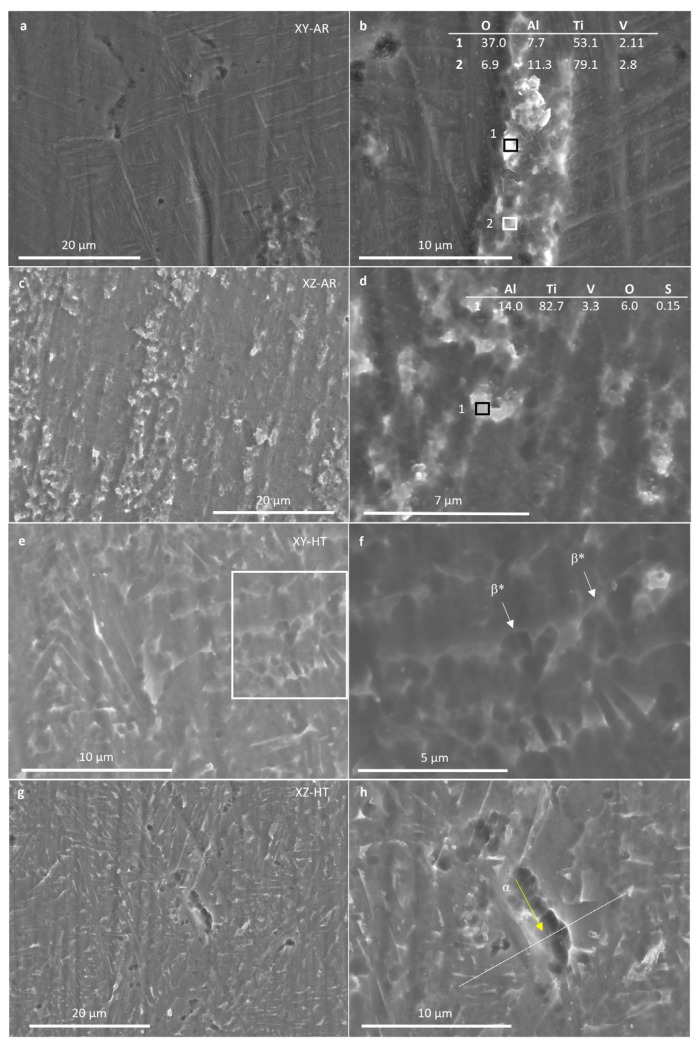

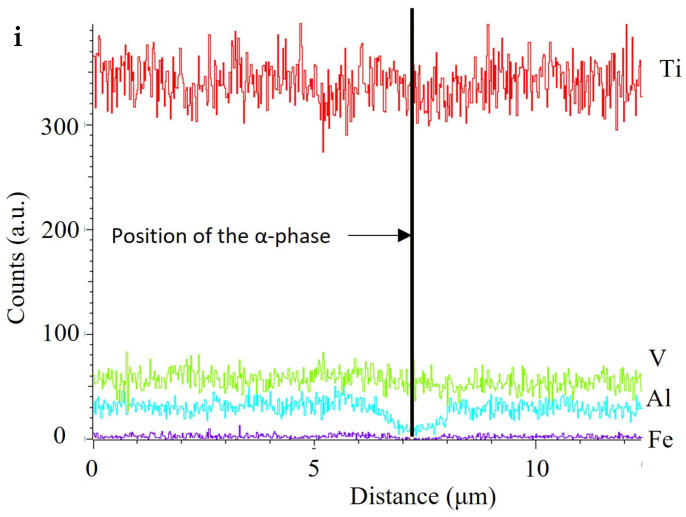
Scanning electron microscopy images (**a**–**h**) and EDS line scan (**i**) of additive manufacturing samples XY-AR (**a**,**b**), XZ-AR (**c**,**d**), XY-HT (**e**,**f**), and XZ-HT (**g**,**h**) after the polarization in Harr solution at 2.15 V for 1 h. (**i**) The line scan of the location indicated in (**h**). (**i**) Elemental profiles of the line scan indicated in (**h**). Labels stand for: α, Ti alpha phase; β, Ti beta phase; β*, beta aggregates.

Figure 10 presents SEM images of the XY-AR (a and b), XZ-AR (c and d), XY-HT (e and f), and XZ-HT (g and h) samples after the polarization in Harr solution. The XY-AR and XZ-AR samples showed a general dissolution of the martensitic α’ matrix and were accompanied by slight protrusion of the β* aggregates and the formation of a thick oxide film. Regarding the XY-HT and XZ-HT samples, α lamellas were preferentially dissolved, and β* aggregates protruded from the surface. However, preferential dissolution of α lamellas was more severe in XZ-HT, with localized corroded regions (Figure 10h). Figure 10i represents the EDS composition profiles taken along the line scan shown in Figure 10h. It can be seen that at the location of the α phase, the concentration of Al decreased drastically. This correlated with the findings in the anodic region in Figure 7b, where XZ-HT presented a second passive segment, while XY-HT had a single passive segment at lower currents.

Considering that it is the 3.5 wt.% NaCl solution where the AM-ed alloys were most impaired, the behavior of one of them (i.e., AMXY-HT) was further studied by EIS tests in this medium at different polarization potentials with respect to the OCP (0, 0.5, 2, and 3 V), using the C alloy as a reference.

#### 3.2.3. Electrochemical Impedance Spectroscopy

**Figure 11:**
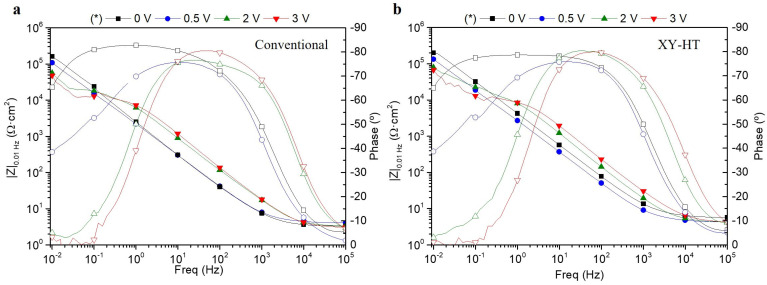
Bode plots recorded for the conventional (**a**) and AM alloy (**b**) during EIS at polarization voltages of 0, 0.5, 2, and 3 V (* with respect to the OCP) in 3.5 wt.% NaCl. These data were fitted with the equivalent circuit shown in Figure 12. Filled symbols correspond to |Z|_0.01 Hz_ (left axis), whereas open symbols correspond to phase angle values (right axis).

Electrical characteristics of the specimens at different voltages (0 V, 0.5 V, 2 V, and 3 V) were considered in order to underpin the degradation mechanism. Figure 11 illustrates Bode plots for conventional (a) and AMXY-HT (b) alloys at the mentioned polarization potentials. Both alloys present similar values in the total impedance modulus measured at the minimum frequency (|Z|0.01 Hz), being that the parameter is known to provide an estimation of the overall corrosion resistance. In addition, both alloys show the same tendency on the |Z|0.01 Hz, with the applied voltage characterized by a decrease in the total impedance down to the range between 5 × 10^4^ and 7 × 10^4^ Ω·cm^2^ at higher voltages. The impedance response also reveals the presence of different relaxation processes. The passive layer is responsible for the high-frequency relaxation process (10^3^ Hz), whereas the middle/low-frequency response of the system (10^1^–10^2^ Hz) can be attributed to the electrochemical activities of the substrate. Regarding the phase angle frequency plots (represented with open symbols), both materials indicate a capacitive behavior at 0 V that changes to a more complex response for higher voltages.

**Figure 12:**
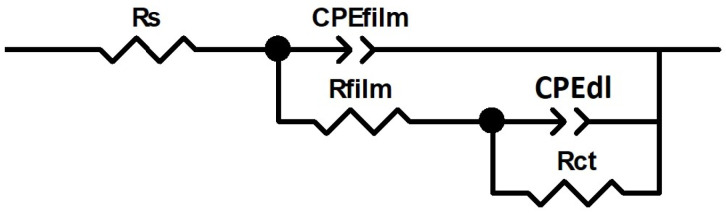
An equivalent circuit used to fit the EIS data.

The equivalent circuit used for fitting the electrical parameters is shown in Figure 12. Constant Phase Elements (CPE) were used instead of capacitances in order to account for the non-ideal behavior of the system. CPE_film_/R_film_ corresponds to the capacitive and resistive behavior of the passive film, and the corrosion process can be described by the double layer capacitance on the electrolyte/metal interface (CPE_dl_) and the charge transfer (R_ct_). Representative results of the fitting (CPE, n, R) are gathered in Table 1. Notice that the first column presents the actual potential values at which the samples were polarized and the last one presents the thickness (d) of the passive film for each condition, calculated following the formula:

$$d\;=\frac{\varepsilon_{0}\cdot \;\varepsilon }{C_{\mathrm{film}}}$$

where ε_0_ and ε are the permittivity of free space and the dielectric constant of the passive layer: 8.85 × 10^−14^ F/cm and 3.97 × 10^1^, respectively. The capacitance of the film (C_f_) was calculated as follows:

Cfilm=CPEfRS+Rct−1+Rfilm−11−nfilm1nfilm

The thickness of the passive layer (Table 1) was slightly higher for the XY-HT sample, and both materials showed a growth of the passive layer with the polarization potential due to the anodic polarization. However, thicker passive film did not lead to better corrosion behavior in the case of the XY-HT sample, which was probably due to the presence of defects. It is revealed that for all the ranges, the applied voltage of the conventional alloy showed slightly higher values of R_film_ and R_ct_, indicating its better corrosion behavior compared with the AM Ti-6Al-4V alloy. In fact, the electrochemical response analyzed by EIS showed different tendencies compared to the conventional material, depending on the fabrication method and heat treatments.

Polarization potentials of 2 and 3 V correspond to the second passive region observed in Figure 4a (from 1.5 to 2.6 V), where the passive current density of the AM alloy was higher than the one of the C alloys. The EIS fitting results at these potentials revealed that the R_film_ of the AM alloy was significantly lower in comparison to the C alloy, despite having similar thicknesses (~13–15 nm, respectively).

## 4. Conclusions

The electrochemical results obtained by PDP in 3.5 wt.% NaCl revealed that the corrosion performance of the additively manufactured alloy, regardless of the heat treatment and building orientation, was worse than that of the conventional alloy. Therefore, the present results indicated that an equiaxial α + β microstructure is more resistant in saline solution than microstructures consisting of α/α’ lamellas and aggregates of the V-rich phase (β*) at the lamella grain boundaries. This suggests that longer heat treatments might be beneficial for AM-ed alloys when immersed in saline media since these would promote larger β phase grains.

Electrochemical impedance spectroscopy at potentials in the OCP region (0–0.5 V with respect to the OCP) and the re-passivation region (2–3 V with respect to the OCP) suggested that despite being thicker, the oxide films formed on the XY-HT sample were less resistant than on the C sample. The most evident microstructure difference is the finer grain size of the AM alloy with respect to the C alloy. It has been discussed that Ti alloys with a refined grain size and a high population density of grain boundaries tend to present increased corrosion resistance [33]. However, this was suggested for alloys produced by severe plastic deformation and does not seem to be the case for the AM alloy of the present study in 3.5 wt.% NaCl.

In general, the behavior of the AM-ed alloys in Harrison’s solutions was comparable to or better than that of the C alloy, particularly for the XY-HT alloy, which revealed slight dissolution of the α phase. The other AM-ed alloys showed greater dissolution of the α/ α’ phases, resulting in protrusion of the β* aggregates and regions with thicker oxide film. Therefore, it is evident that in Harrison’s solution, there is a noticeable influence of building orientation and heat treatment. In the latter case, the elimination of the acicular α’ martensite is particularly beneficial.

The passive behavior of the XY-HT and XZ-HT samples was superior to that of the C alloy in acidified Harrison’s solutions (pH 4) with and without fluorides. This suggested a negative effect of the coarse β phase under these conditions.

## Figures and Tables

**Table 1:** EIS data resulting from fitting the EIS data with the equivalent circuit shown in Figure 12. * OCP + polarization potential.

Substrate	E * (V_Ag/AgCl_)	R_s_ (kΩcm^2^)	CPE_f_ (μSs^n^cm^−2^)	n_f_	R_film_ (kΩcm^2^)	CPE_dl_ (μSs^n^cm^−2^)	n_dl_	R_ct_ (kΩcm^2^)	C_film_	d (nm)
C	−0.4	9.5	1.74	0.92	110	8.18	0.92	1.53	1.1 × 10^−5^	3.49
	0.1	12.6	1.96	0.88	30	9.02	0.86	5.81	7.18 × 10^−6^	4.90
	1.6	9.6	7.85	0.90	896	9.68	0.97	3.56	4.51 × 10^−6^	7.79
	2.6	9.6	4.33	0.94	149	1.22	0.94	3.33	2.67 × 10^−6^	13.18
AM	−0.4	10.1	1.65	0.88	22	9.38	0.87	1.85	5.66 × 10^−6^	6.21
	0.1	9.8	1.91	0.88	13	1.22	0.87	4.84	5.97 × 10^−6^	5.89
	1.6	7.8	7.13	0.93	61	2.49	0.92	2.61	4.7 × 10^−6^	8.63
	2.6	7.8	3.60	0.95	49	1.81	0.93	2.64	2.23 × 10^−6^	15.75

## Data Availability

Data are contained within the article.
